# Expression of somatostatin receptors in oncocytic (Hürthle cell) neoplasia of the thyroid

**DOI:** 10.1038/sj.bjc.6690251

**Published:** 1999-03

**Authors:** L E Tisell, H Ahlman, B Wängberg, L Kölby, M Fjälling, E Forssell-Aronsson, J Mölne, O Nilsson

**Affiliations:** Department of Surgery, Sahlgrenska University Hospital, Göteborg, Sweden; Department of Nuclear Medicine, Sahlgrenska University Hospital, Göteborg, Sweden; Department of Radiation Physics, Sahlgrenska University Hospital, Göteborg, Sweden; Department of Pathology, Sahlgrenska Unversity Hospital, Göteborg, Sweden

**Keywords:** indium-111-DTPA-D-Phe^1^-octreotide, scintigraphy, somatostatin receptors, Hürthle (oncocytic) cell tumour, thyroid

## Abstract

Ten consecutive patients with Hürthle cell lesions of the thyroid (nodule/adenoma/carcinoma) were studied by ^111^In-DTPA-D-Phe^1^-octreotide scintigraphy. Octreotide scintigraphy localized the primary Hürthle cell tumour in eight patients as distinct areas of increased uptake of radionuclide. Two patients with Hürthle cell carcinoma, previously thyroidectomized, had their metastases visualized by octreotide scintigraphy. Northern analyses showed expression of multiple somatostain receptor subtypes. Visualization of the Hürthle cell tumour may be due to a higher expression of somatostatin receptors in the lesions than in surrounding normal thyroid tissue. The tissue/blood ^111^In concentration ratios for tumour samples from five patients showed clearly higher values than observed for normal connective tissue, muscle or lymph nodes. A relatively high uptake of ^111^In was also observed in goiter tissue, which may lead to misinterpretations. The main indication for octreotide scintigraphy in patients with Hürthle cell carcinoma is suspicion of metastatic disease. © 1999 Cancer Research Campaign


*British Journal of Cancer* (1999) **79**(9/10), 1579–1582
© 1999 Cancer Research Campaign
Article no. bjoc.1998.0251

### Expression of somatostatin receptors in oncocytic (Hürthle cell) neoplasia of the thyroid

**LE Tisell[1], H Ahlman[1], B Wängberg[1], L Kölby[1], M Fjälling[2], E Forssell-Aronsson[3], J Mölne[4] and O Nilsson[4]**

Departments of [1]Surgery, [2]Nuclear Medicine, [3]Radiation Physics, and [4]Pathology, Sahlgrenska University Hospital, Göteborg, Sweden

**Summary** Ten consecutive patients with Hürthle cell lesions of the thyroid (nodule/adenoma/carcinoma) were studied by $^{111}$In-DTPA-D-Phe$^1$-octreotide scintigraphy. Octreotide scintigraphy localized the primary Hürthle cell tumour in eight patients as distinct areas of increased uptake of radionuclide. Two patients with Hürthle cell carcinoma, previously thyroidectomized, had their metastases visualized by octreotide scintigraphy. Northern analyses showed expression of multiple somatostain receptor subtypes. Visualization of the Hürthle cell tumour may be due to a higher expression of somatostatin receptors in the lesions than in surrounding normal thyroid tissue. The tissue/blood $^{111}$In concentration ratios for tumour samples from five patients showed clearly higher values than observed for normal connective tissue, muscle or lymph nodes. A relatively high uptake of $^{111}$In was also observed in goiter tissue, which may lead to misinterpretations. The main indication for octreotide scintigraphy in patients with Hürthle cell carcinoma is suspicion of metastatic disease.

**Keywords:** indium-111-DTPA-D-Phe$^1$-octreotide; scintigraphy; somatostatin receptors; Hürthle (oncocytic) cell tumour; thyroid

Neuroendocrine tumours, including thyroid tumours such as medullary thyroid carcinoma (MTC), have been shown to express somatostatin receptors (*sstr*) and can thus be diagnosed by scintigraphic techniques utilizing a radiolabelled somatostatin analogue, $^{111}$In-DTPA-D-Phe$^1$-octreotide (Baudin et al, 1996; Tisell et al, 1997). Five different subtypes of *sstr* have been cloned and shown to be G-protein coupled receptors with seven transmembrane regions. *Sstr*2–3 and *sstr*5 differ from *sstr*1 and *sstr*4 regarding amino acid homology and pharmacological profile. Octreotide binds with highest affinity to *sstr*2 and with lower affinity to *sstr*3 and *sstr*5, while somatostatin binds to all subtypes with high affinity (cf. Bruns et al, 1994). In patients with differentiated thyroid (non-MTC) tumours distant metastases can sometimes be diagnosed and treated with radioiodine. However, one third of these tumours do not accumulate radioiodine (Maxon et al, 1990). Hürthle (oncocytic) cell lesions (nodule/adenoma/carcinoma) belong to this category. The diagnosis of Hürthle cell lesions is today based upon preoperative cytology, or histopathological examination of surgical specimens, demonstrating oncocytic cell features.

The aims of the present study were to compare the outcome of $^{111}$In-DTPA-D-Phe$^1$-octreotide scintigraphy with clinical findings in patients with Hürthle cell lesions of the thyroid and to study the expression of *sstr* in these lesions by Northern blotting using subtype-specific probes. Furthermore, the $^{111}$In concentrations in tumour or normal tissue (Ti) and blood (B) were determined.

### MATERIALS AND METHODS

### Patients

During a 3-year period (1995–97) 10 patients (seven women and three men, mean age 68, range 37–86 years) underwent thyroid surgery with subsequent histological demonstration of Hürthle cell lesions. All lesions were reexamined by two pathologists (ON and JM) and classified according to strict criteria (Rosai et al, 1990) as Hürthle cell nodules ($n$ = 2), Hürthle cell adenoma ($n$ = 4) and Hürthle cell carcinoma ($n$ = 4) (Table 1). Eight of these patients had palpable thyroid tumours, where preoperative fine needle aspiration indicated Hürthle cell neoplasia (seven patients) or follicular thyroid neoplasia (one patient). Two patients had previously been treated for Hürthle cell carcinoma and had metastatic disease (patient nos V and IX). Patient no. V had local symptoms and underwent palliative resection, while patient no. IX had no further treatment. All patients underwent scintigraphy with $^{111}$In-octreotide, and eight patients were also investigated by conventional thyroid scintigraphy (cf. below) prior to surgery (Table 1). $^{111}$In concentrations were determined in both tumour and normal tissues from five patients (Figure 1). Tumour biopsies from four patients (I–IV, two adenomas, one nodule and one carcinoma, cf. Table 1) were investigated by Northern analysis and compared with biopsies of histologically normal thyroid tissue from the contralateral lobe of two patients, who underwent hemithyroidectomy for benign adenomas.

### Scintigraphy

Ten patients received i.v. 210–230 MBq $^{111}$In-DTPA-D-Phe$^1$ octreotide (10–20 µg) (Mallinckrodt, The Netherlands). The amount of $^{111}$In bound to octreotide was higher than 98%. Imaging was done 24–48 h after injection of the radiopharmaceutical. A gamma camera (GE 400 AC/T), equipped with a medium energy parallel hole collimator and connected to a GE STARCAM computer system, was used. Data acquisition was done in a dual window setting (173 and 247 keV). Single photon emission computed tomography (SPECT) of the thoracic cage was performed in all patients (cf. Ahlman et al, 1994). Six patients also underwent thyroid scintigraphy using $^{99m}$Tc-pertechnetate (75 MBq) and the two patients with distant metastases were scanned with

*Received 14 July 1998*
*Revised 10 October 1998*
*Accepted 29 September 1998*

*Correspondence to:* LE Tisell

**Table 1** Results of scintigraphy in patients with Hürthle cell tumours

| Patient | Age (years) and sex | Tumour type | $^{99m}$Tc | $^{111}$In |
|---|---|---|---|---|
| I | 86 F | Carcinoma | Not done | Hot |
| II | 83 F | Nodule | Cold | Hot |
| III | 72 F | Adenoma | Cold | Hot |
| IV | 46 M | Adenoma | Cold | Hot |
| V | 74 F | Carcinoma dist met | No uptake[a] | Hot |
| VI | 50 F | Adenoma | Cold | Hot |
| VII | 37 M | Carcinoma | Cold | Hot |
| VIII | 78 F | Adenoma | Cold | Hot |
| IX | 75 M | Carcinoma dist met | No uptake[a] | Hot |
| X | 78 F | Nodule | Not done | Hot |

[a]Scintigraphy with $^{131}$I.

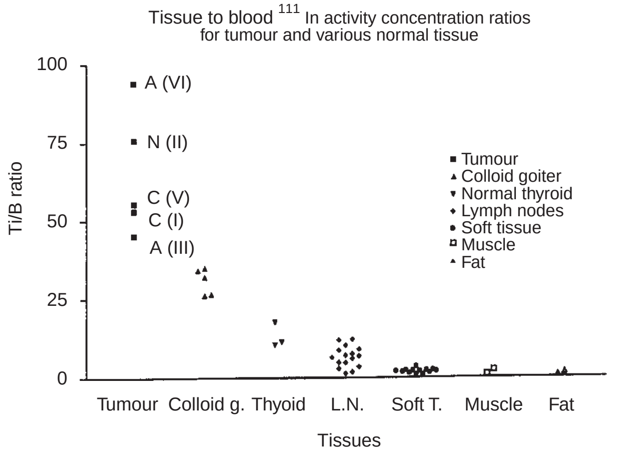

**Figure 1** Tissue to blood (Ti/B) $^{111}$In activity concentration ratios for Hürthle cell lesions of the thyroid (N = nodule, A = adenoma and C = carcinoma) in comparison with various normal tissues. All normal tissues had much lower values than the Hürthle cell lesions and colloid goiter. No evident differences were seen between malignant and benign lesions. Roman numerals refer to patients presented in Table 1

$^{131}$I (100 MBq). These two patients with previous thyroidectomy (nos V and IX) were on thyroxin substitution, which was maintained during $^{111}$In-DTPA-D-Phe$^{1}$-octreotide scintigraphy, but discontinued for the radioiodine examination.

### Measurement of $^{111}$In concentration in tissue samples and blood

Surgery was performed 5–7 days after injection of $^{111}$In-DTPA-D-Phe$^{1}$-octreotide. The surgical specimens from tumour and normal tissues and blood samples, taken during surgery, were weighed and the $^{111}$In concentrations were measured in a calibrated gamma counter equipped with a NaI(T1) well crystal (diameter 7.6 cm, length 7.6 cm, Harshaw, The Netherlands) and a single-channel pulse-height analyser (Elscint, Israel). Corrections were made for background activity and radioactive decay. Tissue-to-blood $^{111}$In concentration ratios (Ti/B), were calculated for biopsied tissues, which were subsequently sent for histopathological examination (cf. Forssell-Aronsson et al, 1995). Tumour biopsies were included in the analysis, if the biopsy contained more than 80% tumour tissue. Nonthyroid specimens (lymph nodes, muscle, soft tissue and fat) were included, if they were tumour-free and contained less than 20% of adherent tissue.

### Northern analyses of *sstr* 1–5 mRNA expression

Fresh tumour specimens were rapidly frozen in liquid nitrogen. RNA was prepared by acid guanidine thiocyanate–phenol–chloroform extraction. Samples of total RNA (20 µg) were heat-denatured and electrophoresed in a 1% agarose gel with 2.2 mol $l^{-1}$ formaldehyde, 1 mmol $l^{-1}$ EDTA, 5 mmol $l^{-1}$ sodium acetate and 20 mmol $l^{-1}$ morpholine propane sulphonic acid (pH 7.0) as running buffer. RNA was transferred to positively charged nylon membranes (Boehringer, Mannheim, FRG) using a vacuum transfer system (Hybaid, Middlesex, UK) and crosslinked to the membrane by UV-light (Stratalinker, Stratagene, La Jolla, CA). Membranes were hybridized in rotating flasks at 65°C (Hybaid, Middlesex, UK) using $^{32}$P-labelled antisense RNA probes. The following probes were used: (1) a 1.126 bp PCR fragment of the human *sstr*1 gene corresponding to nucleotides 352–1478 (Yamada et al, 1992*a*); (2) a 1.7 kb Bam HI/Hind III cDNA fragment of the human *sstr*2 (Yamada et al, 1992*a*); (3) a 1.9 kb Nco/Hind III cDNA fragment of the human *sstr*3 (Yamada et al, 1992*b*); (4) a 2.0 kb Nae I/Xbal cDNA fragment of the human *sstr*4 (Rohrer et al, 1993); (5) a 1.6 kb Eco R1/Sal III cDNA fragment of the human *sstr*5 (Yamada et al, 1993), and (6) a 982 bp fragment of human G3PDH.

Specific labelling was detected after 3–6 days exposure on an imaging plate, followed by reading in a Phosphor Imager (Molecular Dynamics, Sunnyvale, CA).

### RESULTS

### Scintigraphy

The six patients subjected to $^{99m}$Tc-scintigraphy had thyroid tumours that were cold. In the two patients with previous thyroidectomy no metastases could be identified by $^{131}$I-scintigraphy. $^{111}$In-DTPA-D-Phe$^{1}$-octreotide scintigraphy localized the Hürthle cell lesions as distinct areas of increased uptake of radionuclide in all 10 patients (Figure 2A and B; Table 1). In patient no. V with recurrent metastatic disease site localization was obtained by $^{111}$In-DTPA-D-Phe$^{1}$-octreotide in the right low neck region prior to surgery. Multiple lung metastases were also visualized in this patient as well as in patient no. IX (Figure 2C).

### $^{111}$In concentration in tissue samples

Ti/B ratios of removed Hürthle cell lesions from five patients (Figure 1) showed large interindividual variations (range 45–94), but were clearly higher than the Ti/B ratios observed for normal soft tissue, muscle and fat (range 0.8–3.6). Lymph nodes (all without tumour involvement) had values ranging from 1.4 to 12. Normal thyroid tissue had similar values as lymph nodes (Figure 1). Higher levels were observed in parts of the glands with colloid goiter (range 26–35).

Patient no. III with Hürthle cell adenoma underwent total thyroidectomy with lymph node dissection on clinical suspicion of a carcinoma. The histopathological examination demonstrated a Hürthle cell adenoma in a thyroid gland with areas of colloid goiter. The Ti/B ratio of the adenoma was higher (45) than the Ti/B ratios found in the contralateral (26, 32) and pyramidal (34) lobes

© Cancer Research Campaign 1999

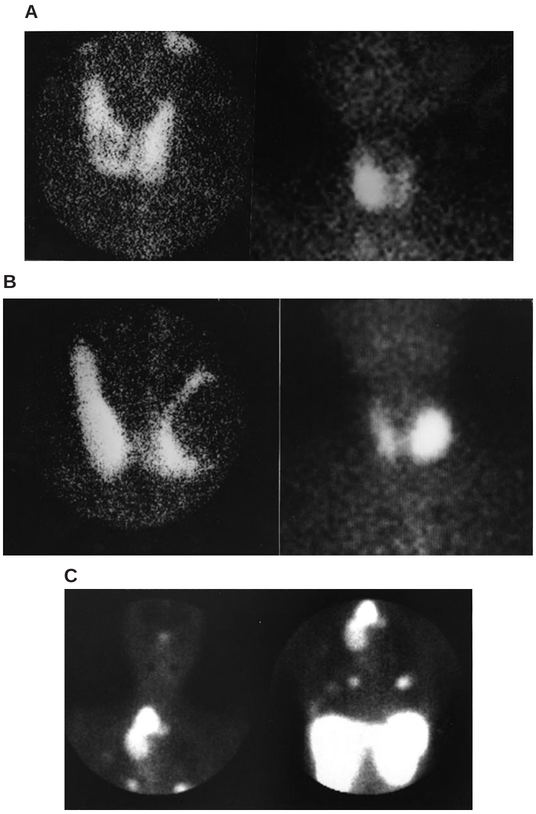

**Figure 2** (**A** and **B**) Anteroposterior views of the thyroid scintigraphically visualized after injection of $^{99m}$Tc-pertechnetate, pinhole collimator (left lane) or $^{111}$In-DTPA-D-Phe$^{1}$-octreotide (right lane). **A** illustrates patient no. VI with a Hürthle cell adenoma in the right lobe and **B** patient no. VII with a Hürthle cell carcinoma in the left lobe. The lesions appeared as cold nodules at $^{99m}$Tc-scintigraphy and demonstrated uptake at octreotide scintigraphy. (**C**) Anteroposterior views of the neck and thorax 24 h after injection of $^{111}$In-DTPA-D-Phe$^{1}$-octreotide in patient no. V with previous thyroidectomy due to Hürthle cell carcinoma. A large tumour mass is visualized in the right lower neck/upper mediastinum besides multiple lung metastases

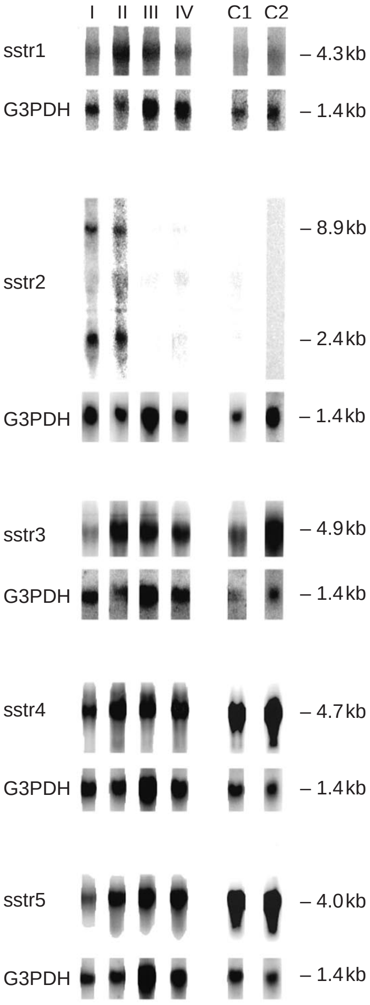

**Figure 3** Northern analysis of *sstr* expression in Hürthle cell lesions with positive octreotide scintigraphy in comparison with normal thyroid tissue. The Hürthle cell lesions expressed 4–5 of the 5 identified *sstr* subtypes. The control tissues showed selective lack of *sstr* 2. The size of mRNA transcripts is indicated to the right. Membranes were rehybridized with G3PDH to verify the integrity of RNA transferred to the membranes. Table 2 is a schematic summary of the results. Roman numerals refer to patients presented in Table 1

with goiter tissue. Fourteen tumour-free lymph nodes were removed with much lower Ti/B values (range 1.4–12).

### Northern analysis

*sstr* mRNA was detected in all Hürthle cell lesions investigated as well as in normal thyroid tissue. Patient no. I with Hürthle cell carcinoma and patient no. II with a Hürthle cell nodule expressed mRNA for all five *sstr*. The two adenoma patients (nos III and IV) had similar receptor-profiles except for a lack of *sstr*2. In the controls the normal thyroid tissue selectively lacked *sstr*2 (Figure 3; Table 2).

**Table 2** Expression of somatostatin receptor subtypes in Hürthle cell lesions and in normal thyroid tissue

| Patient | Tumour type | Expression of somatostatin receptor subtypes | | | | |
|---|---|---|---|---|---|---|
| | | sstr1 | sstr2 | sstr3 | sstr4 | sstr5 |
| I | Carcinoma | + | + | + | + | + |
| II | Nodule | + | + | + | + | + |
| III | Adenoma | + | – | + | + | + |
| IV | Adenoma | + | – | + | + | + |
| Control | Normal thyroid | + | – | + | + | + |
| Control | Normal thyroid | + | – | + | + | + |

*© Cancer Research Campaign 1999*

### DISCUSSION

In a previous study of 21 nonmedullary thyroid carcinomas (papillary, follicular and anaplastic tumour types) $^{111}$In-DTPA-D-Phe$^{1}$-octreotide scintigraphy visualized specific uptakes in all patients with primary tumours and in 75% of the metastatic lesions. The examination gave additional information to radioiodine scintigraphy, especially with regard to skeletal metastases. However, radioiodine seemed to be superior in detecting pulmonary metastases (Postema et al, 1996). To our knowledge, octreotide scintigraphy has not previously been evaluated for Hürthle cell lesions of the thyroid.

In this study 10 patients with Hürthle cell lesions were subjected to octreotide scintigraphy. The primary lesions, or distant metastases, were in each case localized as distinct areas of increased $^{111}$In uptake. Eight patients were investigated by other scintigraphic techniques ($^{99m}$Tc; $n$ = 6 and $^{131}$I; $n$ = 2). In six patients subjected to $^{99m}$Tc-scintigraphy the thyroid lesion appeared as a cold nodule. In our series two patients with previous thyroidectomy due to Hürthle cell carcinoma had their pulmonary metastases revealed by $^{111}$In-DTPA-D-Phe$^{1}$-octreotide scintigraphy, but not by $^{131}$I scanning. Using double-phase scintigraphy with $^{99m}$Tc-sestamibi (MIBI) Vattimo et al (1995) reported visualization of primary Hürthle cell tumours. In many centres double-phase MIBI scintigraphy may serve as the first alternative in the evaluation of cold thyroid nodules or metastases. However, octreotide scintigraphy has the advantage that outcome of surgery can be assessed by one single preoperative injection of $^{111}$In-DTPA-D-Phe$^{1}$-octreotide also used for postoperative imaging (Ahlman et al, 1994). It is important to evaluate the future role of octreotide scintigraphy in patients with Hürthle cell lesions in a prospective study of scintigraphically cold nodules and to investigate whether radiation therapy mediated via *sstr* can be an alternative in disseminated disease (cf. Wängberg et al, 1997). One advantage with both octreotide- and MIBI-scintigraphy is that thyroid substitution therapy does not have to be withdrawn before the examinations.

The visualization of Hürthle cell lesions by octreotide scintigraphy is most likely due to a higher number of *sstr* in the lesions compared with normal thyroid tissue. The receptor-subtype profiles may also influence the visualization, because octreotide has different affinities for the various *sstr*-subtypes (Bruns et al, 1994). Very similar receptor profiles were observed in normal thyroid and tumour tissues by Northern blotting, which indicates that octreotide imaging in these thyroid tumours is not due to a change in receptor profile, but rather to an increased number of *sstr*. A differential diagnostic problem is illustrated by patient no. III with a relatively high uptake of $^{111}$In in nontumour thyroid tissue. The uptake of $^{111}$In in the colloid goiter tissue may represent binding to thyreocytes and lymphocytes. There was no obvious difference between the uptake in Hürthle cells of nodules, adenomas or carcinomas. Uptake of $^{111}$In in the thyroid in benign conditions (endemic colloid goiter, thyroid autonomy and Grave's disease) has been reported previously (Becker et al, 1995). The relative high uptake of $^{111}$In in colloid goiter tissue may lead to misinterpretations. The main indication for octreotide scintigraphy in patients with Hürthle cell tumour is therefore suspicion of metastatic disease.

### ACKNOWLEDGEMENTS

This work was supported by The Swedish Cancer Society (3427, 3911), The Swedish Medical Research Council (5220), The Assar Gabrielsson Research Foundation, IB & A Lundberg Research Foundation, The Swedish Society of Medicine, The Swedish Society for Medical Research, The Göteborg Medical Society, Konung Gustav V's Jubileumsfond, Sahlgrenska Hospital Research Funds, Jubileumsklinikens Cancer Research Funds, Gunvor and Josef Anérs Stiftelse, Axel Linders Stiftelse, Gunnar, Arvid and Elisabeth Nilssons Cancerstiftelse.

### REFERENCES

Ahlman H, Wängberg B, Tisell LE, Nilsson O, Fjälling M and Forssell-Aronsson E (1994) Clinical efficacy of octreotide scintigraphy in patients with midgut carcinoid tumors and evaluation of intraoperative scintillation detection. *Br J Surg* **81**: 1144–1149

Baudin E, Lumbroso J, Schlumberger M, Leclere J, Giammarile F, Gardet P, Roche A, Travagli JP and Parmentier C (1996) Comparison of octreotide scintigraphy and conventional imaging in medullary thyroid carcinoma. *J Nucl Med* **37**: 912–916

Becker W, Schrell U, Buchfelder M, Hensen J, Wendler J, Gramatzski M and Wolf F (1995) Somatostatin receptor expression in the thyroid demonstrated with $^{111}$In-octreotide scintigraphy. *J Nucl Med* **34**: 100–103

Bruns C, Weckbecker G, Raulf F, Kaupmann K, Schoeffer P, Hoyer D and Lübbert H (1994) Molecular pharmacology of somatostatin-receptor subtypes. *Ann NY Acad Sci* **773**: 138–146

Forssell-Aronsson E, Fjälling M, Nilsson O, Tisell LE, Wängberg B and Ahlman H (1995) Indium-111-activity concentration in tissue samples after intravenous injection of indium-111-DTPA-D-Phe$^{1}$-octreotide. *J Nucl Med* **36**: 7–12

Maxon HR and Smith HS (1990) Radioiodine-131 in the diagnosis and treatment of metastatic well-differentiated cancer. *Endocrinol Metab Clin North Am* **19**: 685–718

Postema PTE, de Herder WW, Reubi JC, Oei HY, Kwekkeboom DJ, Bruining HJ, Bonjer J, van Toor H, Hennemann G and Krenning EP (1996). Somatostatin receptor scintigraphy in non-medullary thyroid cancer. *Digestion* **57**: 36–37

Rohrer L, Raulf F, Bruns C, Buettner R, Hofstaedter F and Schule R (1993) Cloning and characterization of a fourth somatostatin receptor. *Proc Natl Acad Sci USA* **90**: 4196–4200

Rosai J, Carcangive ML and DeLellis RA (1990) Tumors of the thyroid gland. *Atlas of Tumor Pathology*. Third series, Fascicle 5. Armed Forces Institute of Pathology, Washington DC, USA

Tisell LE, Ahlman H, Wängberg B, Hansson G, Mölne J, Nilsson O, Lindstedt G, Fjälling M and Forssell-Aronsson E (1997) Somatostatin receptor scintigraphy in medullary thyroid carcinoma. *Br J Surg* **84**: 543–547

Vattimo A, Bertelli P, Cintorino M, Burroni L, Volterrani D and Vella A (1995) Identification of Hürthle cell tumor by single-injection, double-phase scintigraphy with technetium-99m-sestamibi. *J Nucl Med* **36**: 778–782

Wängberg B, Nilsson O, Johansson V, Kölby L, Forssell-Aronsson E, Andersson P, Fjälling M, Tisell LE and Ahlman H (1997) Somatostatin receptors in the diagnosis and therapy of neuroendocrine tumors. *The Oncologist* **2**: 50–58

Yamada Y, Post SR, Wang K, Tager HS, Bell GI, Seino S (1992*a*) Cloning and functional characterization of a family of human and mouse somatostatin receptors expressed in brain, gastrointestinal tract and kidney. *Proc Natl Acad Sci USA* **89**: 251–255

Yamada Y, Reisine T, Law SF, Ihara Y, Kubota A, Kagimoto S, Seino M, Seino Y, Bell GI and Seinos S (1992*b*) Somatostatin receptors an expanding gene family: cloning and functional characterization of human SSTR3, a protein coupled to adenyl cyclase. *Mol Endocrinol* **6**: 2136–2142

Yamada Y, Kagimoto S, Kubota A, Yasuda K, Masuda K, Someya Y, Ihara Y, Li Q, Imura H, Seino S and Seino Y (1993) Cloning, functional expression and pharmacological characterization of a fourth (SSTR4) and a fifth (SSTR5) human somatostatin receptor subtype. *Biochem Biophys Res Commun* **195**: 844–852

 © Cancer Research Campaign 1999
